# Utilization of commercial collagens for preparing well-differentiated human beta cells for confocal microscopy

**DOI:** 10.3389/fendo.2023.1187216

**Published:** 2023-05-25

**Authors:** Brianna R. Brennecke, USeong Yang, Siming Liu, Fatma S. Ilerisoy, Beyza N. Ilerisoy, Aditya Joglekar, Lucy B. Kim, Spencer J. Peachee, Syreine L. Richtsmeier, Samuel B. Stephens, Edward A. Sander, Stefan Strack, Thomas O. Moninger, James A. Ankrum, Yumi Imai

**Affiliations:** ^1^ Department of Internal Medicine, Carver College of Medicine, University of Iowa, Iowa City, IA, United States; ^2^ Fraternal Order of Eagles Diabetes Research Center, University of Iowa, Iowa City, IA, United States; ^3^ Roy J. Carver Department of Biomedical Engineering, University of Iowa, Iowa City, IA, United States; ^4^ Department of Neuroscience and Pharmacology, Iowa Neuroscience Institute, University of Iowa, Iowa City, IA, United States; ^5^ Central Microscopy Research Facility, Roy G. and Lucille A. Carver College of Medicine, University of Iowa, Iowa City, IA, United States; ^6^ Medical Service, Endocrinology Section, Iowa City Veterans Affairs Medical Center, Iowa City, IA, United States

**Keywords:** type IV collagen, type V collagen, CNA35, mitochondria, lipid droplets

## Abstract

**Introduction:**

With technical advances, confocal and super-resolution microscopy have become powerful tools to dissect cellular pathophysiology. Cell attachment to glass surfaces compatible with advanced imaging is critical prerequisite but remains a considerable challenge for human beta cells. Recently, Phelps et al. reported that human beta cells plated on type IV collagen (Col IV) and cultured in neuronal medium preserve beta cell characteristics.

**Methods:**

We examined human islet cells plated on two commercial sources of Col IV (C6745 and C5533) and type V collagen (Col V) for differences in cell morphology by confocal microscopy and secretory function by glucose-stimulated insulin secretion (GSIS). Collagens were authenticated by mass spectrometry and fluorescent collagen-binding adhesion protein CNA35.

**Results:**

All three preparations allowed attachment of beta cells with high nuclear localization of NKX6.1, indicating a well-differentiated status. All collagen preparations supported robust GSIS. However, the morphology of islet cells differed between the 3 preparations. C5533 showed preferable features as an imaging platform with the greatest cell spread and limited stacking of cells followed by Col V and C6745. A significant difference in attachment behavior of C6745 was attributed to the low collagen contents of this preparation indicating importance of authentication of coating material. Human islet cells plated on C5533 showed dynamic changes in mitochondria and lipid droplets (LDs) in response to an uncoupling agent 2-[2-[4-(trifluoromethoxy)phenyl]hydrazinylidene]-propanedinitrile (FCCP) or high glucose + oleic acid.

**Discussion:**

An authenticated preparation of Col IV provides a simple platform to apply advanced imaging for studies of human islet cell function and morphology.

## 1 Introduction

Type 1 and type 2 diabetes results from the loss of functional beta-cell mass due to autoimmune attack or nutritional stress in those with genetic susceptibility (1, 2). Under diabetogenic stress, adaptive and maladaptive responses occur in beta cells within the endoplasmic reticulum, mitochondria, lysosomes, and other organelles, which are often associated with alterations in organelles’ mass, shape, dynamics, and macromolecule distribution (3–6). In addition to organelles, perturbations in cytoskeletal structure are implicated in beta-cell failure in diabetes (7). Thus, morphological assessment of beta cells and surrounding cells at the subcellular level is important to understand beta-cell physiology and the pathogenesis of beta-cell failure in diabetes. Technical advancement in high-resolution fluorescent microscopy and the development of various fluorescent markers now offer an opportunity to acquire a wealth of information regarding changes in the morphological and functional status of cells in response to stimuli and disease conditions. However, the application of high-resolution fluorescent microscopy to human primary beta cells has been limited due to the difficulty in preparing cells on a glass surface amenable to high-resolution imaging. Dispersed human beta cells do not spread well on a glass surface (8), show poor survival after dispersion (9), and undergo epithelial-to-mesenchymal transition quickly on a tissue culture dish (10).

*In vivo*, human beta cells reside in an islet that is a three-dimensional aggregate composed of endocrine cells, non-endocrine cells (e.g., endothelial cells, pericytes), and extracellular matrix (ECM) (11). In addition to cell–cell contact, beta cells depend on the interaction with the ECM for survival and normal function represented by glucose-stimulated insulin secretion (GSIS) (12). Within human islets, ~15% of the surface of each beta cell directly interacts with the ECM in the form of a basement membrane that lies between beta cells and capillaries (12). The basement membrane provides structural support and serves as the scaffold for growth factors including fibroblast growth factor (FGF) and vascular endothelial growth factor (VEGF) (11). In addition, attachment to the ECM activates integrin receptors and other signal pathways that trigger the formation of focal adhesion complexes and determine the polarity of beta cells (13, 14). Human islets have a double-layered basement membrane in contrast to mouse islets that have a single-layer basement membrane (15). As the production of the ECM proteins by beta cells is limited, endothelial cells and pericytes are primarily responsible for the production of basement membrane ECM for an intact islet *in vivo* (12, 16). Considering the importance of the ECM and its loss during the dispersion of human islets, coating a glass surface with ECM components is a logical approach to improving the survival and function of human beta cells seeded on a glass surface (14, 17). As a major advancement, Phelps et al. recently formulated a protocol to prepare a human islet cell monolayer on a glass surface that is suitable for confocal and super-resolution microscopy using commercially available type IV collagen (Col IV) and culturing in neuronal medium (8). In addition to its simplicity, the protocol showed greater attachment of cells compared with laminin, polyornithine, and the ECM from HTB-9 cells. Additionally, human beta cells maintained a highly differentiated status represented by a mono-hormonal expression of insulin and a nuclear expression of NKX6.1/PDX1 for over 4 days in culture (8). Studies that adopted this protocol also reported glucose-responsive [Ca^2+^]_i_, oxygen consumption, and insulin secretion, collectively supporting the preservation of beta-cell functions using the protocol (18, 19). Interestingly, human islet cells in the protocol formed a sheet of cells maintaining cell–cell contact that may also contribute to the preservation of beta-cell function (8).

With highly promising results reported for the new protocol (8, 18, 19), we aim to address adaptability of the protocol to type V collagen (Col V). Being the most abundant collagen in human islets (20) and a key component of the basement membrane, Col IV has been extensively studied and reported to support the survival, maturity, and functionality of beta-cell lines, primary islet cells, and stem cell-derived beta-like cells (15). However, other islet-associated collagens could impact beta-cell health, but they have not been studied for their effect on human beta cells as a glass-coating material. Col V is a low-abundance fibril-forming collagen. Col V is enriched in the islet ECM and critical for beta-cell function; however, it is lost during the isolation process and only minimally reproduced by beta cells (16, 21–23). It has three isoforms that differ with respect to the composition of the three alpha chains that make up the triple helix: α1(V)_2_α2(V), α1(V)_3_, or α1(V)α2(V)α3(V) (20, 21). α1(V)_2_α2(V) affects the geometry and strength of collagen fibers, and mutations of COL5A1 and COL5A2 are associated with the classic Ehlers-Danlos syndrome (20). COL5A3 is found in adipose tissue, human placenta, and pancreatic islets (21, 24). Whole-body deletion of *Col5a3* in mice caused glucose intolerance due to insulin resistance and the reduction of islet mass and insulin secretion (21). The addition of Col V to Matrigel promoted differentiation of human pluripotent stem cells (iPSCs) into islet-like organoids and enhanced glucose-responsive insulin and glucagon secretion from organoids (22). Col V was also shown to increase insulin secretion in INS1 cells (25). However, the effects of Col V on human beta-cell attachment, function, and differentiation are currently unknown.

Here, we compared two commercial preparations of Col IV and one preparation of Col V as coating materials for preservation of beta-cell function and morphology of human islet cells. Col V was as effective as Col IV for culturing well-differentiated beta cells with robust GSIS. However, after extensive assessment of morphology, Col IV was superior in creating human islet cell monolayers with limited cell stacking as compared with Col V. These data highlight the advantage of Col IV as a platform for confocal microscopy. Alarmingly, one Col IV preparation contained little Col IV when assessed by mass spectrometry and collagen-binding adhesion protein (CNA35). When human islet cells were cultured and imaged on well-authenticated Col IV, confocal imaging successfully revealed dynamic changes in two organelles (i.e., mitochondria and lipid droplets (LDs)) in beta and alpha cells.

## 2 Materials and methods

### 2.1 Coating of glass surfaces with collagens and seeding of human islet cells

A 7-mm-diameter glass bottom confocal dish (MatTek Life Sciences) was coated with 50 μg/ml of Col IV (C5533 and C6745 from Sigma) and Col V (C3675 from Sigma) diluted in Hanks’ Balanced Salt Solution (HBSS) with Ca^2+^ and Mg^2+^ at 37 °C for 2 h. After washing with 1.5 ml of HBSS for 15 min four times, each dish was left to dry under UV light for 2 h prior to cell seeding. Institutional Review Board at University of Iowa deemed that experiments using human islets are not a human study. Human islets from non-diabetic organ donors with reported viability and purity above 80% were obtained from the Integrated Islet Distribution Program, Alberta Diabetes Institute or Prodo Laboratories INC (**Supplementary Table 1**) and cultured overnight upon arrival in CMRL 1066 medium with 1% human serum albumin, 1% L-glutamate, and 1% penicillin/streptomycin at 37°C and 5% CO_2_ for recovery from shipping. Dispersion, seeding, and culturing of human islet cells basically followed methods published by Phelps et al. with minor modifications (8). In brief, human islets were washed with PBS, incubated with Accutase (SCR005, Millipore Sigma, St Louis, MO) at 37°C for 5 min, dispersed by gentle pipetting, and passed through a 40-μm strainer to make single-cell suspension. The dispersed islet cells were resuspended in 10% HI-FBS CMRL at 140,000 cells/ml. Cell suspension was added to a precoated confocal dish at approximately 35,000 cells/cm^2^. After 6 h of incubation at 37°C and 5% CO_2_, a 2-ml neuronal medium described by Phelps et al. (8) was applied to each dish. After overnight, 3 μM cytarabine (ARA-C) was added to the culture medium to eliminate fibroblast-like cells as originally reported by Phelps et al. (8), and cells were cultured for additional 3 days.

### 2.2 Immunocytochemistry

After removing old culture medium, cells were fixed in a 1:1 mixture of 8% paraformaldehyde and fresh neuronal medium at 37°C for 15 min and stored in 4 °C immersed under PBS until analyses. Cells were permeabilized with 0.1% Triton-X, incubated in a primary antibody mixture prepared in PBS containing 0.1% Triton-X overnight in 4 °C, washed three times for 5 min in PBS, and incubated with secondary antibodies for 1 h at room temperature (RT) in the dark. The antibodies used are shown in **Supplementary Table 2**. After washing secondary antibodies with PBS once for 5 min and with water twice for 3 min, nuclei were visualized by adding 1 μg/ml 4′,6-diamidino-2-phenylindole (DAPI, from Thermo Fisher) for 3 to 5 min. DAPI was removed by washing with water once for 3 min and with PBS twice for 5 min. After removal of excess liquid, cells were covered with 180 μl of prolong mounting solution and an 18 × 18 mm coverslip was placed over cells.

### 2.3 Assessment of cell identity, nuclei number in z-axis of islet cluster, and cell area

Cells immunostained with insulin (INS), glucagon (GCG), and NKX6.1 were analyzed using a Zeiss 710 microscope via a 63× oil lens to acquire z-stack images at an interval of 0.3 µm covering the entire height of each cell cluster. Imaris analysis software (Oxford Instruments) was used to create a surface for INS and GCG positive cells. For nuclei too close for the software to accurately differentiate, a “spot” was manually placed in the center of each nucleus. A mask of the spots was then created leaving behind saturated spheres in the center of each cell as the nuclear surface. Then, the machine learning component of Imaris was used to automatically identify the following criteria: INS^+^NKX^+^, INS^+^NKX^-^, INS^+^GCG^+^, GCG^+^NKX^+^, GCG^+^NKX^-^, INS^-^GCG^-^NKX^-^. For each new donor, three to five images were tested for correct identification visually. For counting the number of nuclei in the Z-axis, Imaris software was utilized to obtain a 3D visual of cluster geometry focusing on nuclear DAPI staining. The XZ and YZ orthogonal planes were used to determine the maximum number of nuclei in the Z-axis for each cluster.

For determining the beta-cell border, human islet cells were immunostained with NTPDase 3, Syntaxin 1, and NKX6.1. A Zeiss 980 microscope Airy Scan 2 via a 63× oil lens in an airy scan mode was utilized to acquire z-stack images at an interval of 0.15 µm covering the entire height of an islet cell cluster. Imaris software was used to create a 3D image, and an XY plane was scanned from the bottom to select the plane that contains the maximum footprint of beta cells for each cell cluster. The drawing tool in ImageJ was used to outline individual beta-cell borders to measure the perimeter and area of a beta-cell and nucleus.

### 2.4 Area and number of cell clusters

The DAPI signal of cell clusters on collagens was captured using a Leica DM6B epifluorescent microscope at 20× air objective with a focus at the base of each cluster to capture the cells directly adhered to the coated surface. As cells tend to attach more toward the center of the dish, we systematically captured three images from the innermost 2-mm-diameter circle of the dish. An additional three images between a 2- and 4-mm-diameter circle of the dish were captured when more than three fields are analyzed. The acquired images were analyzed by ImageJ (see **Supplementary Methods**, macro cell adhesion area) to measure the number of clusters and the area of each cluster.

### 2.5 SDS-polyacrylamide gel electrophoresis

Collagens were denatured in 4× NuPAGE LDS sample buffer (NP0007, Invitrogen) with 20 μM DTT at 80 °C for 10 min and resolved on 4%–12% gradient NuPAGE Bis-Tris Plus Gels (NP0326BOX, Invitrogen). The PAGE gels were stained using Pierce Silver Stain for Mass Spectrometry (24600, Thermo Scientific) following the manufacturer’s instructions. Affinity of proteins with collagen-binding adhesion protein 35 (CNA35) conjugated with Alexa Fluor 488 (CNA35) (26, 27) was tested by incubating gels with CNA35 at 1:4,000 in PBS with 0.5% Tween 20 (PBST) for 120 min at RT followed by washing in water for 24 h. The CNA35 plasmid was provided as a kind gift from Dr. Magnus Hook at Texas A&M Health. Green fluorescence was imaged by iBright (Invitrogen). C6745 resolved on SDS-polyacrylamide gel electrophoresis (SDS-PAGE) gels was also transferred to nitrocellulose membranes, incubated with anti-rabbit IgG-HRP antibody (SC2357, Santa Cruz at 1:2,000) at 4 °C overnight, and visualized with ECL Western Blotting Substrate (32106, Fisher Scientific) on a Kodak IM2000 Imager.

### 2.6 Dot blot

The concentration of different commercial collagens was adjusted to 0.3 µg/µl, and the indicated amount of collagen was pipetted onto a nitrocellulose membrane as an individual dot. The membrane was dried in air for 30 min and subsequently washed once with PBST. Then, the membrane was incubated with CNA35 at 1:2,000 in PBST at RT for 50 min in the dark. Afterward, the membrane was washed three times for 15 min with PBS. The fluorescent signals on the membrane were imaged using an iBright FL1500 Imaging System (Invitrogen) under fluorescent mode.

### 2.7 GSIS

Human islet cells cultured for 5 days on coating materials were preincubated for 1 h in Krebs–Ringer buffer (KRB) containing 0.25% BSA and 2.8 mM glucose. Thereafter, cells were sequentially incubated for 1 h each with KRB containing 2.8 and 16.8 mM glucose. After washing, human islet cells were solubilized in RIPA buffer (Sigma) containing protease inhibitors and stored at -20°C for total insulin measurement. Insulin-secreted and insulin contents were measured with STELLUX Chemiluminescent Human Insulin ELISA kit (ALPCO) according to the manufacturer’s protocol.

### 2.8 Total internal reflection fluorescent microscopy

Human islet cells fixed with 4% paraformaldehyde were stained for INS, E-cadherin, and DAPI as in Section 2.2. Following the post-staining washes, 1.5 ml PBS was added for aqueous imaging media. Cells were imaged using a Leica total internal reflection fluorescent (TIRF) AM microscope 100× oil objective in TIRF mode with a penetration depth of 100–150 nm. TIRF images of insulin granules were analyzed using a series of ImageJ macros (see **Supplementary Methods**, TIRF macro). TIRF images of E-cadherin were used first to create the region of interest (ROI) eliminating any background outside the cell cluster. The local auto threshold (Bernsen method, https://imagej.net/plugins/auto-local-threshold) measured insulin granules with varying intensities. The Bernsen method was chosen as there was a great deal of variation within insulin granule pixel intensity. This method allowed the selection of both dim and bright granules. Insulin intensity within each granule was measured and allocated into background (0–29), low (30–100), medium (101–179), and high (180–255) intensities. Granules with insulin intensity less than 30 or granule area greater than 0.5 µm were excluded from the data.

### 2.9 Morphometry of mitochondria and LDs in human islet cells treated with FCCP and high glucose + oleic acid loading

Human islet cells were cultured on glass surfaces coated by collagen preparations as above. Cells were transduced with CellLight Mitochondria-GFP, BacMam 2.0 (Thermo Fisher), at 50 particles per cell (PPC) 2 days before harvest. 2 μM Bodipy 558/568 C12 (Bodipy C12) was added during the last 16 h of culture to visualize triglyceride-rich LDs (28). A part of human islet cells was cultured in neuronal medium containing 20 mM glucose and 0.5 mM oleic acid (OA) for the last 48 h of culture. FCCP was added at 2.5 μM for the last 6 h of culture.

After fixation, beta cells and alpha cells were immunostained as in Section 2.2. Zeiss 980 microscope Airy Scan 2 in an airy scan SR mode was utilized to acquire z-stack images via a 63× oil lens at an interval of 0.15 µm covering the entire height of islet cell cluster. Two sets of 5 consecutive slices of Z-stack image were selected to represent the bottom half and top half of each islet cluster. Five to 10 islet clusters were analyzed for each condition. Thereafter, five consecutive slices were merged into one image via the maximum-intensity projection plugin in ImageJ/Fiji. Projection images thus created were assessed for mitochondrial morphology using pre-programmed macro script in ImageJ/Fiji (1.53v, NIH) (29, 30). Briefly, images were converted to binary images of mitochondrial particles and automated morphometry of mitochondrial particles was performed to obtain mitochondrial length and form factor (perimeter^2^/(4π × area)) (29, 30). The size and number of LDs were measured as published (31). The area of beta cells (INS^+^) was also obtained by ImageJ/Fiji.

### 2.10 Statistics

Data are presented as mean ± SEM unless otherwise stated in the figure legends. Differences of numeric parameters between two groups were assessed with Student’s t-tests. Welch’s correction was applied when variances between two groups were significantly different by F test. Multiple group comparisons used one-way ANOVA with a *post-hoc* test as indicated. Prism 9 (GraphPad, La Jolla, CA) was used for statistical tests unless specified otherwise. p < 0.05 was considered significant.

## 3 Results

### 3.1 Human beta cells cultured on commercial Col IV and -V preparations all show high nuclear localization of NKX6.1

**Figure 1:**
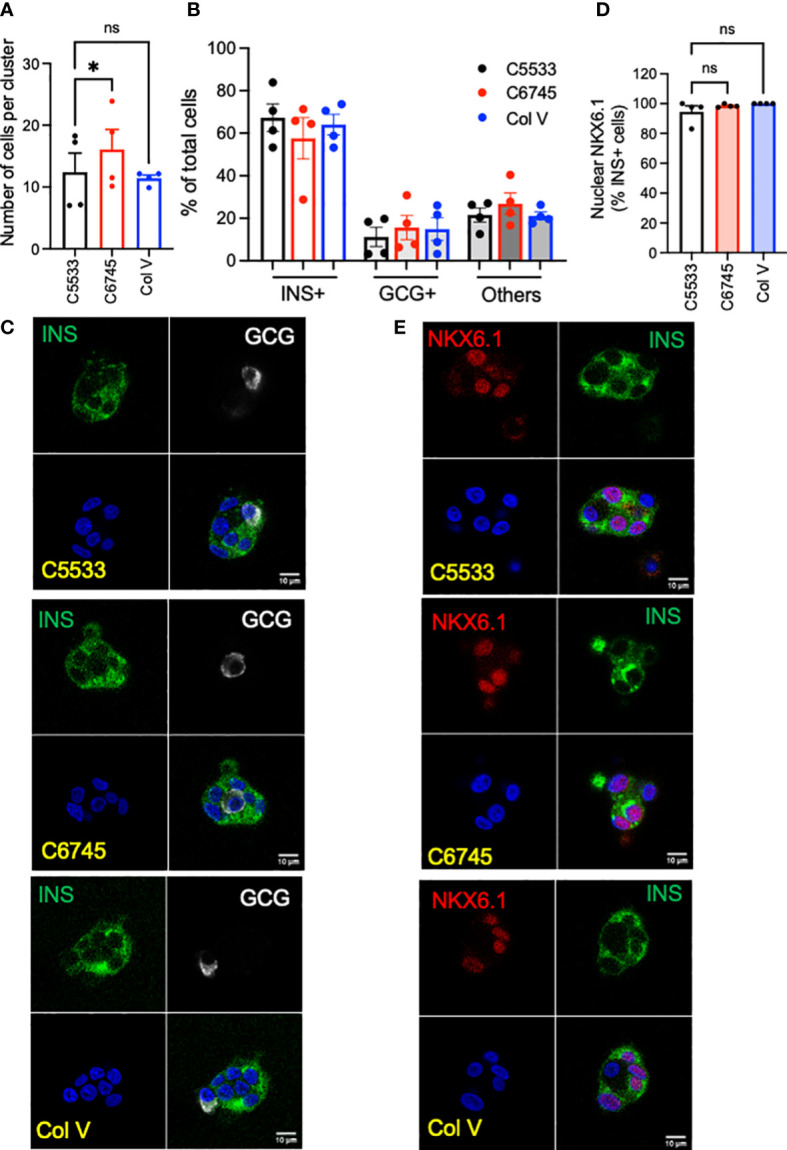
Single-cell suspension of human islet cells was seeded on glass surfaces coated with type IV collagen 5533 (C5533), type IV collagen 6745 (C6745), and type V collagen (Col V) and cultured for 5 days. **(A)** The number of nuclei per an islet cell cluster and **(B)** the proportion of INS^+^ and GCG^+^ cells were counted after staining for insulin (INS, green), glucagon (GCG, white), and DAPI (blue). **(C)** Representative images. **(D)** Human islet cells prepared as in **(A)** and stained for INS (green), NKX6.1 (red), and DAPI (blue) and % of INS+ cells with nuclear NKX6.1 was measured. **(E)** representative images. Data are mean ± SEM. n = 4 donors. Scale bar, 10 μm. Statistics by RM one-way ANOVA. n.s.; not significant, *p < 0.05.

We tested three commercially available collagen preparations, two Col IV (C5533 from human placenta and C6745 from coculture of fibroblasts and epithelial cells) and one Col V (from human placenta) to coat cover glasses for confocal imaging. After 5 days of culture, single-cell suspension of human islet cells formed islands containing ~10 cells on average on a cover glass coated with Col IV and Col V preparations (**Figure 1A**). The islet cluster on C6745 contained slightly higher numbers of cells than C5533, whereas the number of cells in clusters on Col V was equivalent to C5533 (**Figure 1A**). The proportion of INS^+^ and GCG^+^ cells in islet clusters was similar for all three collagen preparations with beta- to alpha-cell ratios around 3:1, slightly higher than the reported beta- to alpha-cell ratio in adult human islets (**Figures 1B, C**) (32). In all three collagen preparations, INS^+^ cells showed close to 100% positivity of nuclear NXK6.1, indicating that beta cells maintained a well-differentiated status on all three collagen preparations (**Figures 1D, E**). Non-beta cell staining of NKX6.1 was negligible (data not shown).

### 3.2 Morphology of islet cluster and beta-cell spreading differ between two commercial Col IV preparations

**Figure 2:**
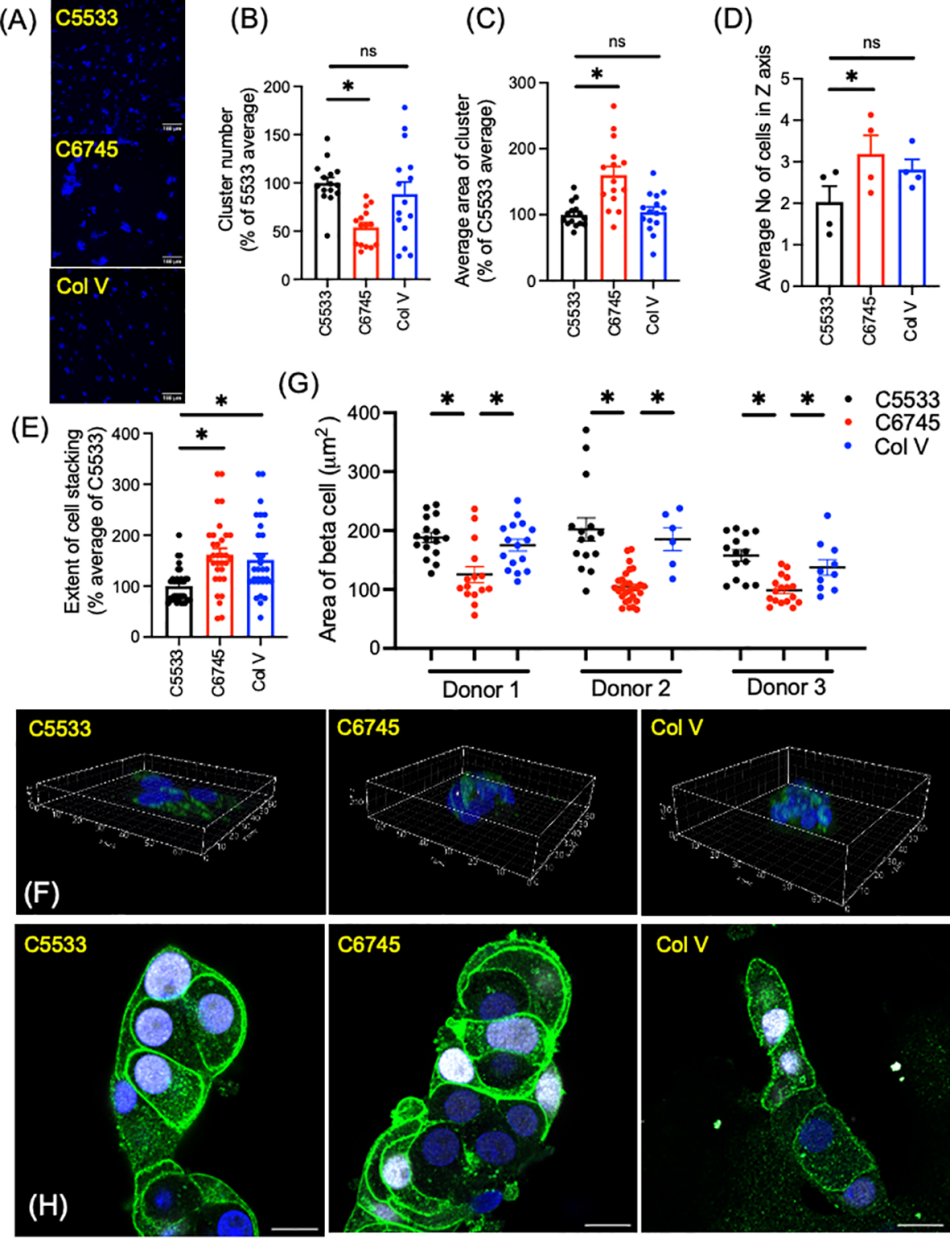
Dispersed human islet cells seeded and cultured on type IV collagen 5533 (C5533), type IV- collagen 6745- (C6745), and type V collagen- (Col V) coated glass surfaces were visualized by anti-insulin antibody (green) and DAPI (blue). **(A)** Representative 20× objective epifluorescence microscope image showing DAPI (scale bar, 100 μm). **(B)** The number of clusters and **(C)** cell cluster area in 0.44 mm^2^ field were counted and expressed as an average for C5533 in each of five donors as 100%. n = 3 fields per donor × 5 donors total 15. **(D-F)** Z-stack images of islet cell clusters on each collagen surface was captured via a confocal microscope and processed by Imaris software to count the number of nuclei along the Z-axis for each cluster **(D)** average number of nuclei for each donor (n = 4 donors). **(E)** data expressed taking average of C5533 as 100% for each donor (n = 8 clusters per donor × 4 donors = 32). **(F)** 3D representative images (axis in μm, see **Supplementary Video 1**). **(G, H)** Beta-cell border was visualized by a mixture of anti-NTPDase3 and syntaxin 1 antibodies (green). Beta-cell identity was confirmed by nuclear expression of NKX6.1 (white). Beta-cell area was measured as in methods in three donors. n = 6 to 29 cells. Each dot represents one cell. Data are mean ± SEM. Statistics by RM one-way ANOVA. n.s, not significant; *p < 0.05.

While all three collagens were similar in the proportion of INS^+^ cells and high positivity of nuclear NKX6.1 in beta cells, we noted significant differences in the number and size of islet clusters between the two Col IV preparations (C6745 and C5533) when the same number of cells was seeded (**Figure 2A**). In addition to containing slightly lower numbers of cells per cluster (**Figure 1A**), islet cells on C5533 formed more islet clusters (**Figure 2B**, **Supplementary Figures 1A, B**), each with a smaller area of adhesion to the glass surface (**Figure 2C**, **Supplementary Figures 1C, D**) compared with C6745. There was no statistically significant difference between C5533 and Col V. In addition to forming cell clusters with smaller adherent areas on the glass surface, islet cells were stacked less on C5533 compared with C6745 as the number of nuclei found along the Z-axis was lower (**Figures 2D–F**, **Supplementary Video**). The average number of nuclei along the Z-axis in four donors was 2.03 ± 0.38 nuclei for C5533, 3.19 ± 0.45 nuclei for C6745, and 2.81 ± 0.24 nuclei for Col V (mean ± SEM, **Figure 2D**).

Previously, Phelps et al. reported that beta cells on Col IV have larger cell areas than those on polyornithine and laminin, indicating Col IV attachment results in flatter beta cells (8). To compare the beta-cell area between these three collagen preparations, we defined the border of beta cells by immunoreactivity to NTPDase 3 and syntaxin 1, two proteins preferentially distributed to the cell membrane in beta cells (**Figures 2G, H**) (33, 34). As shown in **Figure 2H**, beta cells marked by nuclear expression of NKX6.1 showed strong expression of NTPDase 3 and syntaxin 1 at the cell periphery. Although the shape and size of cells varied, beta cells on C5533 and Col V had significantly larger cell areas (**Figure 2G**) and cell area/nuclear area (**Supplementary Figure 1E**) compared with C6745. Collectively, human islet cell clusters on C5533 Col IV preparation formed single to double layers of cells and contained beta cells with large cell areas. Human islet cells plated on Col V showed more cell stacking than those on C5533, but the rest of parameters including cell areas were similar between Col V and C5533.

### 3.3 Col IV contents differ significantly between two commercial preparations

**Figure 3:**
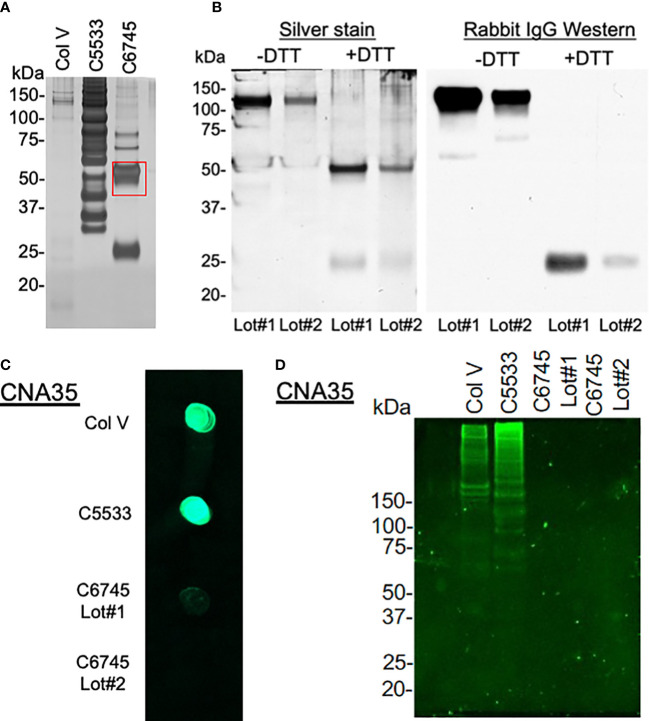
**(A)** 1.5 μg/lane of type IV collagen C5533 (C5533), type IV collagen C6745 (C6745), and type V collagen (Col V) preparations were run on SDS-PAGE under reducing condition and visualized by silver staining. The 50-kDa band of C6745 (red rectangle) was analyzed by mass spectrometry (**Supplementary Figure 2A**). **(B)** Two lots of C6745 (Lot#1 and #2) were separated by SDS-PAGE under non-reducing (-DTT) and reducing (+DTT) conditions and visualized by silver staining (left) or by immunoblot for rabbit IgG (right) after transfer to a nitrocellulose membrane. **(C)** 1.5 μg of C5533, C6745 (Lot#1 and Lot#2), and Col V were applied to a nitrocellulose membrane and incubated with CNA35, as described in the methods. **(D)** 5.7 μg/lane for each collagen preparation was separated by SDS-PAGE and incubated with CNA35, as described in the methods.

As the large differences in attachment behavior between the two Col IV preparations (C6745 and C5533) were puzzling, we analyzed the molecular weight distribution of peptides in the collagen preparations after SDS-PAGE using silver staining (**Figure 3A**). Col V showed faint bands primarily at high molecular weight. C5533 showed variable-size proteins predominantly above 60 kDa, whereas C6745 produced two major bands of 50 and 25 kDa (**Figure 3A**). Liquid chromatography with tandem mass spectrometry (LC-MS/MS) analysis of the 50-kDa band present in C6745 Col IV preparation identified the rabbit IgG heavy chain as the major protein (**Supplementary Figure 2A**). The presence of rabbit IgG in C6745 was not limited to a single lot preparation, as another lot (Lot #2) of C6745 subjected to Western blot also contained rabbit IgG, albeit to a lesser degree than Lot #1 (**Figure 3B**). In addition, untargeted mass spectrometry of C5533 identified fragments of Col IV, whereas these were not detected in C6745, further indicating that C6745 does not contain an appreciable amount of Col IV compared with C5533 (**Supplementary Figure 2B**).

To provide an additional confirmation of the presence or absence of collagens, the affinity of three collagen preparations with fluorescent-tagged collagen-binding adhesion protein CNA35 (35) was tested. A dot blot analysis showed strong binding of C5533 and Col V with CNA35, whereas two separate lots of C6745 had minimum binding (**Figure 3C**). Incubation of SDS-PAGE gel with CNA35 showed that high molecular weight bands in Col V and C5533 interact with CNA35 (**Figure 3D**). Again, C6745 showed minimum binding. Our results indicate that the authentication of collagen preparation is important and CNA35 binding provides a simple lab technique for quick authentication of collagens.

### 3.4 Secretory function of beta cells was similar on three collagen preparations

**Figure 4:**
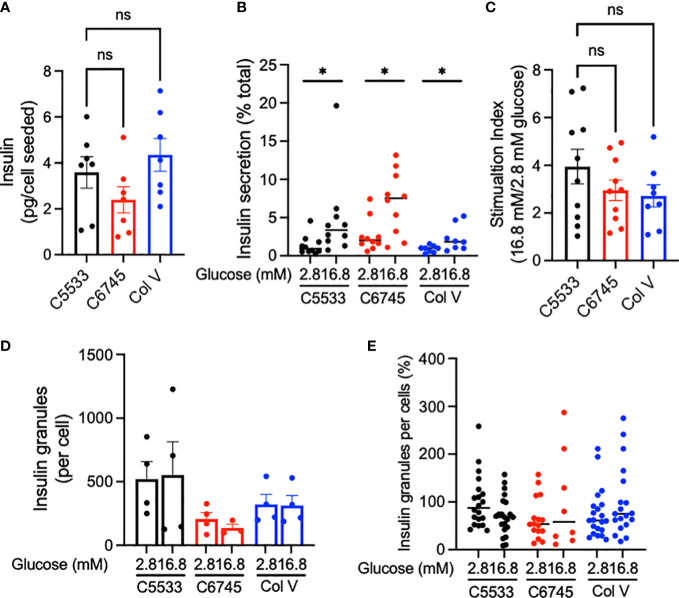
**(A)** Insulin contents and **(B)** insulin secreted at indicated glucose concentrations were obtained from human islet cell clusters cultured for 5 days on type IV collagen C5533- (C5533), type IV collagen C6745- (C6745), and type V collagen- (Col V) coated glass surfaces as described in methods. **(C)** Ratio of insulin secreted at high and low glucose. n = 7 donors **(A)** and 8~10 donors **(B, C)**. **(D, E)** TIRF image was captured for insulin-positive granules on glass surface coated by three coating preparations after incubation in 2.8 or 16.8 mM glucose for 30 min. **(D)** The number of granules per cell for each donor (n = 4). **(E)** Data are expressed taking the average number of granules per cell for cells on C5533 at 2.8 mM glucose in each donor as 100% and data from four donors are combined (n = 8~21). Data are mean ± SEM except for **(B)** and **(E)** that indicate medium. Statistics by RM one-way ANOVA **(A)** or mixed-effect models **(B, C)**. n.s, not significant; *p < 0.05.

Despite differences in attachment behavior (**Figure 2**) and low contents of Col IV in C6745, all three coating materials allowed culturing of well-differentiated INS^+^ cells that maintained nuclear NKX6.1 expression (**Figure 1D**). Thus, we next compared insulin secretion in human islets cultured on three collagen preparations. Cells on C6745 showed a trend toward lower insulin content when corrected for the number of cells seeded compared with those on C5533 (**Figure 4A**, **Supplementary Figure 3A**) that may reflect a difference in number of beta cells attached to the glass surface and/or insulin content per beta cell. GSIS corrected for insulin content tended to be higher for cells on C6745 than those on C5533 (**Figure 4B**). However, the stimulation index of GSIS was comparable for all three preparations including C6745, indicating that beta-cell function was maintained similarly regardless of the type and quality of collagens, concordant with the high nuclear expression of NKX6.1 in INS^+^ cells (**Figure 4C**, **Supplementary Figure 3B**).

Since C5533 flattens INS^+^ cells and increases the area of attachment on the coated surface, we performed TIRF microscopy to compare insulin granules at the attachment surface for three coating materials. The insulin granule number per cell trended from highest to lowest in C5533, Col V, and C6745 at low glucose (**Figures 4D, E**) but did not reach statistical significance. Glucose exposure for 30 min did not result in a statistically significant difference in granule numbers between the three coating materials either (**Figures 4D, E**).

### 3.5 C5533 Col IV preparation allowed imaging of mitochondria and lipid droplets in human beta cells

**Figure 5:**
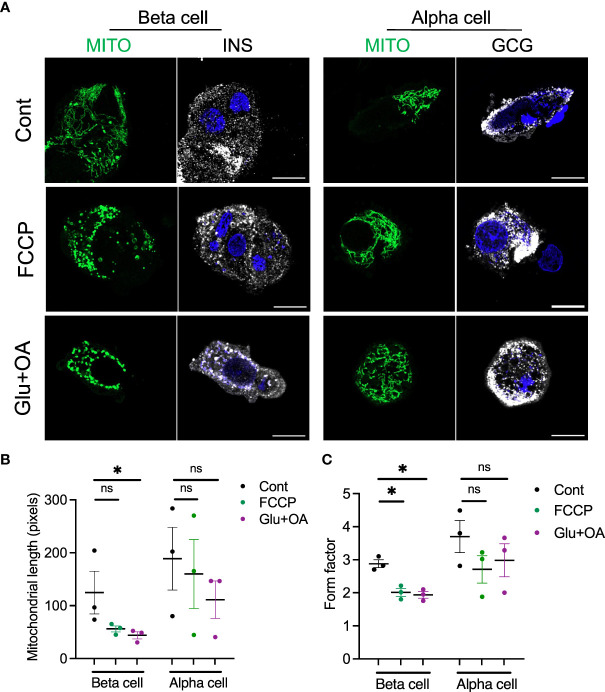
The morphology of mitochondria was assessed in beta and alpha cells plated on C5533 and culture in neuronal medium only or with addition of FCCP or glucose + OA. **(A)** Representative images showing mitochondria-GFP (green), INS or GCG (white), and DAPI (blue). Scale bar, 10 μm. **(B)** Mitochondrial length and **(C)** form factors in beta and alpha cells were obtained in three donors. Each dot represents the mean value of data obtained from 10 to 20 images for each donor (see **Supplementary Figures 3C–F** from which mean values were obtained). Data are mean ± SEM. RM one-way ANOVA with Dunnett’s multiple-comparison test. n.s; not significant, *p < 0.05.

**Figure 6:**
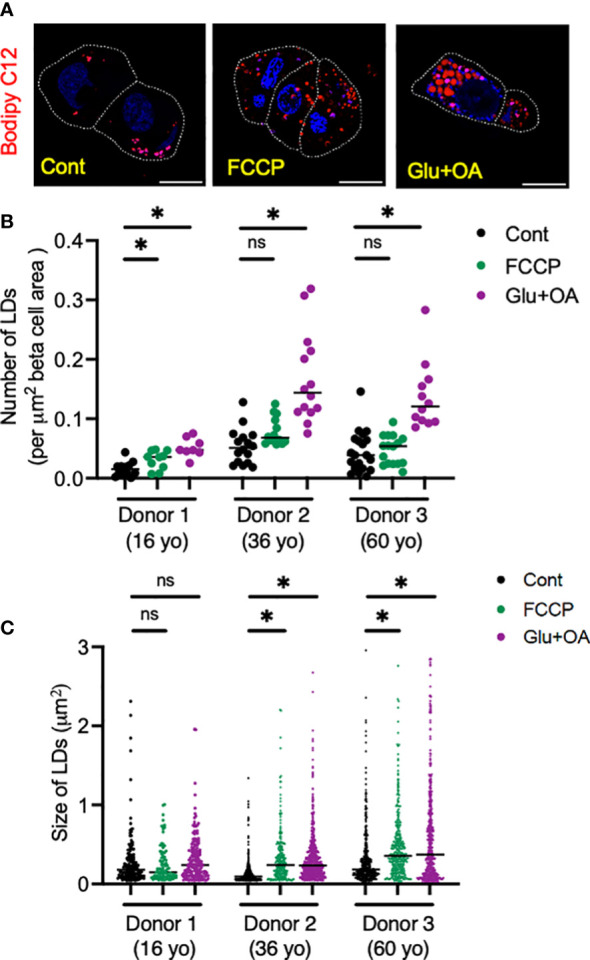
Human islets from three donors aged 16 (donor 1), 32 (donor 2), and 60 (donor 3) year-old (yo) were plated on C5533 and cultured in neuronal medium only or with addition of FCCP or glucose + OA as in methods. LDs were visualized by overnight incubation with Bodipy C12 (red). **(A)** Representative images from donor 2. DAPI in blue. Beta cells are marked by a white line. Scale bar, 10 μm. **(B)** Number of LDs corrected by beta-cell area. n = 8 to 20 images. **(D)** Size of LDs expressed as area (μm^2^). Each dot represents single LD and data combine LDs from 8 to 20 images for each condition. Data are mean ± SEM. Statistics by one-way ANOVA with Dunnett’s multiple-comparison test. n.s; not significant, and *p < 0.05.

Lastly, we tested whether the morphology of two organelles (i.e., mitochondria and LDs) changes in human beta cells on C5533 in response to stimuli known to alter their morphology in a wide range of cells; FCCP is known to cause fragmentation of mitochondria, and nutritional load increases LDs (31, 36). Both FCCP and high glucose + OA shortened the mitochondrial length and decreased the form factor in beta cells, providing evidence that both conditions fragment mitochondria in human beta cells in culture and support the utility of the protocol to obtain morphometric data (**Figures 5A–C**, **Supplementary Figures 3C–F**). Although statistical significance was not reached with three donors, mitochondria in alpha cells tend to be tubular with longer mitochondria and higher form factor than those in beta cells at the baseline. Also, alpha cell mitochondria showed less change after incubation with FCCP or glucose + OA (**Figures 5A–C**). Bodipy C12 is a fluorescent fatty acid analog that is preferentially incorporated into triglyceride-rich LDs (28). Culturing in high glucose + OA for 48 h increased the number of LDs in beta cells in all three donors studied (**Figures 6A, B**). The 6-h incubation with FCCP increased either the number or the size of LDs in all donors, indicating that the impairment in mitochondrial respiration promotes LD accumulation in human beta cells (**Figures 6B, C**). It has been reported that LD formation in beta cells is limited in juvenile beta cells (37). In agreement, we noted that beta cells of donor 1 (16-year-old) had fewer LDs at the baseline and did not increase LD size in response to FCCP or glucose + OA compared with the other two adult donors.

## 4 Discussion

In this study, we compared commercial preparations of Col IV and Col V as coating materials to culture human islet cells on glass surfaces. We noted that the beta-cell phenotype was maintained similarly for all preparations, but preparations using well-authenticated Col IV supported human beta-cell attachment on a glass surface with characteristics advantageous for imaging: less cell stacking and a large cytosolic area. Also, we call attention to the importance of authenticating commercial collagen preparations and report on a simple method for collagen authentication that utilizes fluorescent collagen binding protein CNA35. Lastly, confocal microscopy of human islet cells on Col IV-coated glass allowed to assess responses of mitochondria and LDs to stimuli in human islet cells. Collectively, we provide information that aids application of advanced imaging on human primary islet cells to address pathophysiology of human pancreatic islets.

Col IV and laminin are the major structural constituents of the basement membrane and have been used to attach human beta cells on a glass surface (8, 34). Here, we tested whether non-basement membrane collagen Col V is a beneficial coating material for human islet cells. Col V is present in islet ECM and supports beta-cell mass and function based in a knockout mouse study and studies of iPSC-derived beta cells and INS1 cells (16, 21–23). Here, we found that the differences between Col IV (C5533) and Col V as coating material were relatively small; the functional status of beta cells and the beta-cell area on glass were not significantly different between the two collagens. The major difference we observed between Col IV and Col V was greater cell stacking in islet cluster on glass coated with Col V. This highlights that Col IV is a preferred coating material when cell stacking is not desired.

While variation among donors was evident, human beta cells increased GSIS significantly on all three coating materials to levels comparable with the stimulation index typically seen for human intact islets in culture. Even C6745, which did not contain significant levels of Col IV, allowed the culture of human beta cells with high nuclear expression of NKX6.1. The maintenance of beta-cell features in islet clusters in our study resembles pseudoislets created by reaggregation of dispersed islet cells that preserve the beta-cell phenotype well in culture (38, 39). The maintenance of three-dimensional contact between human islet cells in islet culture likely accounts for the efficacy of three coating materials for the preservation of the beta-cell phenotype. It is encouraging that the flattening of cells and reduction to mono- to double cell layers by C5533 do not impair the beta-cell response to glucose or differentiation, as shown in the current study and by others (8, 19). In addition to coating materials, the culture medium formulated by Phelps et al. (8), which is low in neuroactive amino acids (i.e., L- aspartate, L-glutamate, and L-cysteine) and supplemented with antioxidant-rich B-27, might play an important role in maintaining the differentiated phenotype of human beta cells in prolonged culture.

Commercial preparation of Col IV simplifies its utilization by researchers but apparently requires investigators to perform authentication. Col IV has a highly complex and heterogenous structure in native ECM. Col IV is produced by cells as a rope-like triple helical protomer of three alpha chains for which six subtypes exist (40). Placenta used for the isolation of C5533 predominantly contains the COL4A1 and COL4A2 alpha chains. Col IV protomers secreted from cells undergo extensive multimer formation to create a high molecular mesh in the basement membrane. Isolation of Col IV from biological materials is typically performed by protease digestion that releases a rope-like central triple-helical domain of collagens leaving behind domains (i.e., 7S and NC1) that connect collagen helix to create a high molecular mesh (40). Thus, source materials and methods of isolation can create large variations in both the composition and size of Col IV fragments. Apparently, one preparation (C6745) was heavily contaminated by rabbit IgG when obtained by us, which was not obvious in non-reducing SDS-PAGE. We demonstrated that fluorescently tagged collagen binding protein CNA35 is useful to confirm the presence of collagen. Also, our detailed description of the morphology of human islet cells on Col IV will serve as a reference for the expected behavior of human islet cells on Col IV from human placenta. When seeded at 35,000 cells/cm^2^, human islet cells formed mono-double layers of cell clusters containing ~10 cells.

We have demonstrated the utility of Col IV coating to visualize organelle changes in human primary islet cells by confocal microscopy and obtained several interesting findings. The fragmentation of mitochondria by FCCP and nutritional load appeared to be more prominent in beta cells than in alpha cells. Thus, our data provide proof of principle that human islet cells on Col IV serves as a platform to compare the behavior of beta and alpha cells simultaneously under the same condition when they have physical contact. The increase in the size or number of LDs by FCCP in human beta cells indicates that the impairment in mitochondrial respiration is sufficient to cause LD accumulation in human beta cells. Beta cells of a juvenile human donor failed to expand LD size in response to FCCP and nutritional load. Clearly, further studies are required to confirm findings in larger number of donor islets and to address how age and cell type differentially regulate dynamics of mitochondria and LDs.

This protocol to seed human islet cells on collagen-coated glass is advantageously simple; however, several limitations merit discussion. Dispersion and culturing may alter human islet cell phenotype from the phenotype of intact islets *in vivo*. While these collagens allow flattening of beta cells compared with other materials (8) (Col V in the current study), beta cells on Col IV (C5533) are still more cuboidal than beta-cell lines and rodent beta cells (unpublished data) and show a heterogenous and complex shape (**Figure 2C**). Thus, it is warranted to sample multiple planes of islet cell clusters to capture changes in different subcellular domains of beta cells. Alternatively, multi-photon microscopy enables fluorescent imaging with better penetration and has been used to monitor exocytosis and bioenergetics in intact pancreatic islets (41, 42). With the improvement in spatial resolution, applicability of multi-photon microscopy for organelle imaging has been increasing (43, 44). However, this imaging technique is highly complex and expensive in comparison with confocal microscopy. Also, the permeability of reagents and light scattering remain as potential issues for thick samples. Advancements in electron microscopy provide an additional means to obtain biological information at subcellular levels but also suffer from their own limitations (45). Ultimately, it will be important to combine multiple imaging and non-imaging modalities to address a particular biological question as discussed in a recent review (45).

## Data availability statement

The original contributions presented in the study are included in the article/**Supplementary Material**. Further inquiries can be directed to the corresponding author.

## Author contributions

YI conceived the study and is responsible for all contents of the manuscript. BB (all aspects), UY (all aspects), SL (GSIS and Zeiss 980 imaging), FI (morphometric analysis), BI (morphometric analysis), AJ (morphometric analysis), LK (morphometric analysis), SP (human islet culture), SR (insulin ELISA), SBS (TIRF assay), ES (collagen analysis), SS (mitochondrial morphology), TM (image acquisition by microscopy and image analysis), and JA (morphometric analysis) were responsible for the acquisition and/or analysis of the data. YI and BRB designed the research, drafted the manuscript, and critically revised the manuscript for important intellectual content. All authors revised and approved the final version of the manuscript.
