# Supplementary material for: Utilization of commercial collagens for preparing well-differentiated human beta cells for confocal microscopy
Source: Front Endocrinol (Lausanne). 2023 May 25;14:1187216. doi: 10.3389/fendo.2023.1187216 (PMC10248405; doi:10.3389/fendo.2023.1187216)

The utilization of commercial collagens for preparing well-differentiated human beta cells for confocal microscopy

Brianna R Brennecke[1,2,3], USeong Yang[1,2,3], Siming Liu[1,2], Fatma S Ilerisoy[1,2,3], Beyza N Ilerisoy[1,2,3], Aditya Joglekar[1,2], Lucy B Kim[1,2], Spencer J Peachee[1,2], Syreine Richtsmeier [1,2], Samuel B Stephens[1,2], Edward A Sander[3], Stefan Strack[4], James A Ankrum[2,3], Yumi Imai[1,2, 5*]

*** Correspondence:** Yumi Imai yumi-imai@uiowa.edu

Supplementary methods

Cell adhesion area (ImageJ):

Images of nuclei were acquired at the base of each cluster analyze the area of each cluster at the collagen coating cell interface. Dilate and erode functions were used to connect adjacent nuclei in each cluster.

1. run("8-bit");
2. run("Duplicate...", "duplicate channels=5");
3. setAutoThreshold("Default");
4. //run("Threshold...");
5. setThreshold(30, 255, "raw");
6. //setThreshold(30, 255);
7. setOption("BlackBackground", false);
8. run("Convert to Mask");
9. run("Despeckle");
10. run("Dilate");
11. run("Dilate");
12. run("Dilate");
13. run("Erode");
14. run("Erode");
15. run("Erode");
16. run("Despeckle");
17. run("Analyze Particles...", " circularity=0.10-1.00 display exclude clear add");

Cell Differentiation + Cell Identity/Ratio (IMARIS):

IMARIS analysis software was used to create a surface for each channel. For the nuclei, some were too close together for the software to accurately identify and differentiate so a "spot" was manually placed in the center of each nucleus. A mask of the spots was then created leaving behind saturated spheres in the center of each cell. This new channel is used to create the nuclear surface. The machine learning component of Imaris was used to automatically identify the following criteria: INS+NKX+, INS+NKX-, INS+GCG+, GCG+NKX+, GCG+NKX-, INS-GCG-NKX-. For each new donor, 3-5 images would need to be reevaluated for incorrect identification. The machine learning function allows user input to create criteria and identify objects that fall into each specific identity. The software then records the intensity of the different channels and the distance of those intensities to the specific object. These trends are recorded and corrected until the software is accurately identifying all cells. Once 100% successful, the software can apply the rules to other images.

TIRF (ImageJ):

Macro 1: "Cell Adhesion Area"

1. run("8-bit");
2. run("Duplicate...", "duplicate channels=5");
3. setAutoThreshold("Default");
4. //run("Threshold...");
5. setThreshold(30, 255, "raw");

6. //setThreshold(30, 255);
7. setOption("BlackBackground", false);
8. run("Convert to Mask");
9. run("Despeckle");
10. run("Dilate");
11. run("Dilate");
12. run("Dilate");
13. run("Erode");
14. run("Erode");
15. run("Erode");
16. run("Despeckle");
17. run("Analyze Particles...", " circularity=0.10-1.00 display exclude clear add");

Using the new binary image, the image calculator function in ImageJ was used to subtract the binary image from the original stack (all 5 images). This created the region of interest and eliminated any background not in the cell adhesion area.

Macro 2: “LAT_INS_Granules”
1. run("Arrange Channels...", "new=4");
2. run("Auto Local Threshold", "method=Bernsen radius=2 parameter_1=30 parameter_2=0 white");
3. run("Convert to Mask");
4. run("Watershed");
5. run("Analyze Particles...", "size=0.00-infinity circularity=0.10-1.00 display exclude clear add");

This macro created a mask for all insulin granules.
Macro 3: “ROI Quantification”
1. run("Arrange Channels...", "new=45");
2. roiManager("multi-measure measure_all");

The last macro collects the number, area, min, mean, and max pixel intensity of the granule location for ISN and E-Cadherin.

SDS-PAGE and In-gel Trypsin Digestion:
20 µg of C6745 was loaded on NuPage 4-12% Bis-Tris precast gels (Invitrogen, USA) and separated at 150 V for 50 min. The gel was stained using a Pierce mass spec compatible silver stain kit (Thermo Scientific, USA) following the manufacturer’s directions. In-gel digestion was performed as published with slight modification (1). Briefly, the targeted protein bands from SDS-PAGE gel were manually excised, cut into 1 mm^3 pieces, and washed in 100 mM ammonium bicarbonate:acetonitrile (1:1, v/v) and 25 mM ammonium bicarbonate /acetonitrile (1:1, v/v) to achieve complete destaining. The gel pieces were further treated with acetonitrile, to effectively “dry” the gel segments and then reduced in 50 µL of 10 mM DTT at 56 °C for 60 min. After this, gel-trapped proteins were alkylated with 55 mM Chloroacetamide (CAM) for 30 min at room temperature. The gel pieces were washed with 25 mM ammonium bicarbonate: acetonitrile (1:1, v/v) twice to removed excess DTT and CAM. Then, 50 µL of cold trypsin

solution at 10 ng/µL in 25 mM ammonium bicarbonate was added to the gel pieces and they were allowed to swell on ice for 60 min. Digestion was conducted at 37 °C for 16 h. Peptide extraction was performed three times adding 100 µL of 50% acetonitrile/0.1% formic acid for 0.5 h; combining the supernatants. The combined extracts were concentrated in a lyophilizer and rehydrated in 15 µL of Mobile Phase A (0.1% formic acid with 3% acetonitrile) for LC-MS/MS.

In Solution LysC-Trypsin 2 Step Digestion:
Six micrograms of each protein sample (C5533 and C6745) were reduced and alkylated in 100µl lysis buffer (Lysis buffer: 6 M Guanidinium hydrochloride, GdnHCl), 5 mM tris(2-carboxyethyl)phosphine, TCEP, 10 mM chloroacetamide, CAA, 100 mM Tris–HCl pH 8.5) at 98 °C for 10 min (2). After cooling to room temperature, proteins were digested with LysC (Wako) at the ratio of enzyme-to-protein of 1:30 (w/w) at 37 °C for 2 hours. Samples solution was diluted to a final concentration of 1 M GdnHCl with 25 mM Tris-HCl pH 8.5, then incubated with trypsin at a 1:20 ratio overnight. The next morning, additional trypsin was added to each sample to establish a 1:50 protease to protein ratio (w/w) and digestion continued 3 hours at 37°C. Digested samples were acidified to pH 2-3 with 50% TFA and centrifuged at 20,000 x g for 15 min to pellet insoluble material. The supernatant peptides were desalted with C18 'stage tips' (1, 3). Peptides, eluted in 200 µl 70% ACN and 0.1%FA, were concentrated by lyophilization and reconstituted in 30µL of Mobile Phase A for LC-MS/MS analysis.

Liquid chromatography-tandem mass spectrometry (LC-MS/MS):
In gel tryptic digestion of C6745 and in solution LysC-trypsin 2 step digestion of C6745 and C5533 were analyzed by LC-MS/MS utilizing an Orbitrap Fusion Lumos mass spectrometer (Thermo Fisher Scientific, San Jose, CA) coupled to an Easy-nLC-1200™ System (Proxeon P/N LC1400).
For LC, the fused silica column tapered from 100 µm ID (Polymicro) to ~8 µm at the tip were packed with 2.7 micron Halo C18, MDC particles using a He-pressurized SS cylinder. Extracted peptides were separated by the analytical column (10 cm in length x 100 µm id) at 0.4 µL/min flow rate using a 70 min gradient composed of linear and static segments wherein buffer A is 0.1% formic acid and B is 80%ACN, 0.1% Formic acid. The gradient began first with holds at 4% for 3 min then makes the following transitions (%B, min): (2, 0), (35, 46), (60, 56), (98, 62), (98, 70). Data acquisitions begin with a survey scan (m/z 380 -1800) acquired on a Q-Exactive Orbitrap mass spectrometer (Thermo) at a resolution of 120,000 in the off axis Orbitrap segment (MS1) with Automatic Gain Control (AGC) set to 3E06 and a maximum injection time of 50 ms. MS1 scans were acquired every 3 sec during the 70 min gradient described above. The most abundant precursors were selected among 2-6 charge state ions at a 1E05 AGC and 70 ms maximum injection time. Ions were isolated with a 1.6 Th window using the multi segment quadrupole and subject to dynamic exclusion for 30 seconds if they were targeted twice in the prior 30 sec. Selected ions were then subjected to high energy collision-induced dissociation (HCD) in the ion routing multipole (IRM). Targeted precursors were fragmented by (HCD) at 30% collision energy in the IRM. HCD fragment ions were analyzed using the Orbitrap (AGC 1.2E05, maximum injection time 110 ms, and resolution set to 30,000 at 400 Th). Both MS2 data were recorded as centroid and the MS1 survey scans were recorded in profile mode.

Proteomic Searches:

Initial spectral searches were performed with Byonic search engines (Protein Metrics ver. 2.8.2). Search databases were composed of the Uniprot KB for species 9606 (Human) downloaded 2-8-22 and Swissprot downloaded in March 2021. In either search, an equal number of decoy entries were created and searched simultaneously by reversing the original entries in the target databases. Precursor and fragment mass tolerance was set to 10 ppm respectively. A fixed 57 Da modification was assumed for cysteine residues while a variable Oxidation modifications was allowed at methionine. A variable GG modification at lysine was set to monitor Ubiquitylation with potential phosphorylation accessed at Ser and Thr residues. The False Discovery Rate was maintained at 1% by tracking matches to the decoy database.

Supplementary Table 1
Human islets used for the study

Unique identifier	Donor Age (years)	Donor Sex (M/F)	Donor BMI (kg/m^2)	Donor HbA1c (%)	Origin/source of islets	Figure used
SAMN22818629	45	F	21.7	5.5	IIDP University of Pennsylvania	Fig. 1A, 1B, 1D, 2B, 2C, 2D and 2E Supplementary Fig. 1A-D
R415	32	M	26.6	5.8	Alberta Diabetes Institute	Fig. 1A, 1B, 1D, 1F, 2B, 2C, 2D and 2E Supplementary Fig. 1A-D
R417	48	M	27.5	5.5	Alberta Diabetes Institute	Fig. 1A, 1B, 1D, 2B, 2C, 2D and 2E Supplementary Fig. 1A-D
R421	60	F	25.9	5.4	Alberta Diabetes Institute	Fig. 1A, 1B, 1D, 2B, 2C, 2D and 2E Supplementary Fig. 1A-D
SAMN24579752	55	M	25.2	5.4	IIDP The Scharp-Lacy Research Institute	Fig. 1A, 1C, 1E, 2B, 2C, 4A, 4B, 4C Supplementary Fig. 1A-D, 3A-B
SAMN25690319	49	M	32.5	5.5	IIDP The Scharp-Lacy Research Institute	Fig. 4A-E Supplementary Fig. 3A-B
SAMN25860453	44	M	25.8	6.0	IIDP University of Pennsylvania	Fig. 4A-E Supplementary Fig. 3A-B
SAMN26177826	52	M	25.3	4.2	IIDP The Scharp-Lacy Research Institute	Fig. 4A-E Supplementary Fig. 3A-B
R434	50	F	24.2	5.7	Alberta Diabetes Institute	Fig. 4A-E Supplementary Fig. 3A-B
SAMN27619977	37	M	31.3	5.9	IIDP Southern California Islet Cell Resource Center	Fig. 4A-C Supplementary Fig. 3A-B
SAMN28157682	37	M	30.3	5.0	IIDP Southern California Islet Cell Resource Center	Fig. 4A-C Supplementary Fig. 3A-B
SAMN28501433	48	M	32.3	5.7	IIDP Southern California Islet Cell Resource Center	Fig. 4A-C Supplementary Fig. 3A-B
SAMN29494177	18	M	25.3	5.0	IIDP Southern California Islet Cell Resource Center	Fig. 4B-C
SAMN29494287	54	M	30.8	5.1	IIDP Southern California Islet Cell Resource Center	Fig. 4B-C
SAMN31525423	30	F	39.9	5.3	IIDP Southern California Islet Cell Resource Center	Fig. 2G Supplementary Fig. 1E
SAMN31618441	34	M	25.8	5.0	IIDP University of Wisconsin	Fig. 2G, 2H Supplementary Fig. 1E
22294	36	M	24.4	5.7	Prodo laboratories INC	Fig. 2G Supplementary Fig. 1E
SAMN32641505	16	M	29.6	5.4	IIDP Southern California Islet Cell Resource Center	Fig. 5B,5C, 6B, 6C Supplementary Fig. 3C-F
23044	36	M	34.0	4.9	Prodo laboratories INC	Fig. 5A, 5B,5C, 6A, 6B, 6C Supplementary Fig. 3C-F
23046	60	F	32.9	5.0	Prodo laboratories INC	Fig. 5B,5C, 6B, 6C Supplementary Fig. 3C-F

All donors were non-diabetic.
IIDP: Integrated islet distribution program

Supplementary Table 2

List of antibodies used for the study

	Catalog No	dilution	Figures used
Rabbit anti insulin	Santa Cruz Biotechnology, SC-9168	1:300	Fig. 1B-E, Fig. 2D-E. Fig. 4D-E.<br>Fig. 5A-C, Fig. 6A-C
Mouse anti NKX6.1	Developmental Studies Hybridoma Bank, F55A12	1:100	Fig. 1D-E
Guinea pig anti glucagon	Takara Bio USA, M182	1:300	Fig. 1B-E, Fig. 2D-E, Fig. 5A-C
Mouse anti NTPDase 3	Ectonucleротides-ab, hN3-B3s	1:80	Fig. 2G-H
Mouse anti-syntaxin 1	Santa Cruz Biotechnology, SC-12736	1:50	Fig. 2G-H
Rabbit anti NKX6.1	Abcam, Ab221549	1:300	Fig. 2G-H
Mouse anti cadherin	BD Transduction Laboratories 610181	1:100	Fig. 4D-E
anti-rabbit IgG-HRP antibody	Santa Cruz Biotechnology, SC2357	1:2000	Fig. 3B
Alexa 488 anti rabbit IgG	Invitrogen, A11070	1:300	
Alexa 488 anti mouse IgG	Invitrogen, A11017	1:300	
Alexa 568 anti mouse IgG	Invitrogen, A11019	1:300	
Alexa 647 anti guinea pig IgG	Invitrogen, A21450	1:300	
Alexa 647 anti rabbit IgG	Invitrogen, A21069	1:300	

Figure legends

Supplementary Fig. 1

(A-D) Dispersed human islet cells seeded and cultured on Col IV 5533 (C5533), Col IV 6745 (C6745), and Col V coated glass surfaces were visualized using DAPI. Number (A; C5533 vs. C6745, B; C6745 vs. Col V) and area (C; C5533 vs. C6745, D; C6745 vs. Col V) of clusters in 0.44 mm^2 field were counted for 3 fields for each donor. Average of 3 fields in the same donor is connected by line. n= 4~5 donors. Statistics by student's t test. (E) Beta cell border in human islet cluster was visualized by anti-NTPDase3 and anti-syntaxin 1 antibodies as in Fig. 2H. Beta cell area/nuclear area was measured as in methods in three donors. Each dot represents one cell. n= 6 to 29 cells. Data are mean ± SEM Statistics by RM one-way ANOVA. n.s; not significant, *; $p<0.05$.

Supplementary Fig. 2

(A) Peptides detected by LC-MS/MS of in gel tryptic digest of 50 kDa band in Figure 3A were searched against Swissprot data base as in methods. (B) Peptides detected by LC-MS/MS in solution LysC-Trypsin 2step digestion of Col IV (C5533) are aligned with COL4A2 (UniProtKB:P08572).

Supplementary Fig. 3

(A-B) Glucose-stimulated insulin secretion (GSIS) was performed for human islet cell cluster cultured for five days on Col IV 5533 (C5533), Col IV 6745 (C6745), and Col V coated glass surfaces as described in methods. (A) insulin contents (pg/number of cells seeded) were expressed taking a value for C5533 in each donor as 1 (n=8 donors). (B) stimulation indexes of GSIS for different coating materials were connected by line for each donor (n= 8 donors). (C-F) the morphometry of mitochondria was performed on beta and alpha cells plated on C5533 and culture in neuronal medium only or with addition of FCCP or glucose + OA for three donors. (C) mitochondrial length in beta cells, (D) mitochondrial length in alpha cells, (E) form factor in beta cells and (F) form factor in alpha cells in 10 to 20 images acquired as in methods. Each dot represents one image. Data are mean ± SEM Statistics by RM one-way ANOVA (A) and one-way ANOVA (C-F) all with Dunnett's multiple comparisons test. n.s; not significant and *; $p<0.05$.

References

1. Yu CL, Summers RM, Li Y, Mohanty SK, Subramanian M, Pope RM. Rapid identification and quantitative validation of a caffeine-degrading pathway in Pseudomonas sp. CES. J Proteome Res. 2015;14(1):95-106.
2. Jersie-Christensen RR, Sultan A, Olsen JV. Simple and Reproducible Sample Preparation for Single-Shot Phosphoproteomics with High Sensitivity. Methods Mol Biol. 2016;1355:251-60.
3. Ishihama Y, Rappsilber J, Mann M. Modular stop and go extraction tips with stacked disks for parallel and multidimensional Peptide fractionation in proteomics. J Proteome Res. 2006;5(4):988-94.

Supplementary figure 1

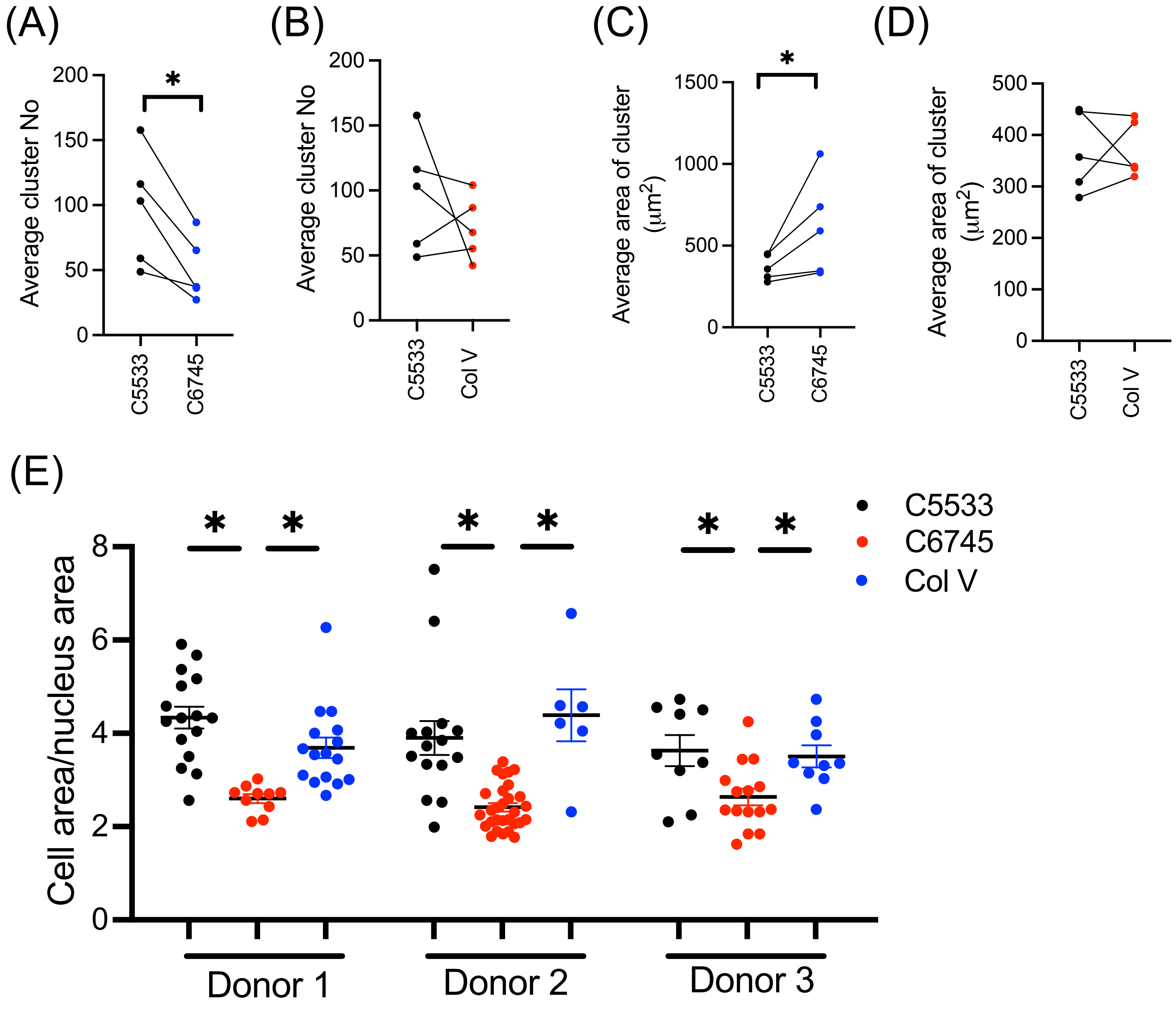

Supplementary Fig. 2

(A)

Protein Rank	Description	|Log Prob|	Best |Log Prob|	Best score	Total Intensity	# of spectra	# of unique peptides	# of mod peptides	Coverage %	# AA's in protein	Protein DB number
1	>sp|P04264|K2C1_HUMAN Keratin, type II cytoskeletal 1 OS=Homo sapiens OX=9606 GN=KRT1 PE=1 SV=6	45.51	5.29	739.40	39841540.4	46	29	3	38.35	644	204133
2	>sp|P35527|K1C9_HUMAN Keratin, type I cytoskeletal 9 OS=Homo sapiens OX=9606 GN=KRT9 PE=1 SV=3	27.99	2.64	511.80	20862390.6	32	26	3	41.25	623	204085
3	>sp|P01870|IGHG_RABIT Ig gamma chain C region OS=Oryctolagus cuniculus OX=9986 PE=1 SV=1	27.12	7.78	809.90	1300073067.6	91	24	10	51.70	323	193899
4	>sp|P13645|K1C10_HUMAN Keratin, type I cytoskeletal 10 OS=Homo sapiens OX=9606 GN=KRT10 PE=1 SV=6	19.32	2.39	476.60	10269943.2	18	18	1	27.57	584	203993
5	>sp|P35908|K22E_HUMAN Keratin, type II cytoskeletal 2 epidermal OS=Homo sapiens OX=9606 GN=KRT2 PE=1 SV=2	13.33	2.60	493.40	5159661.0	10	10	0	16.59	639	204124
6	>sp|P01826|HV1A_RABIT Ig heavy chain V-A1 region BS-5 OS=Oryctolagus cuniculus OX=9986 PE=1 SV=1	3.54	2.78	384.60	94132241.8	8	2	0	6.90	116	188478
7	>sp|P02533|K1C14_HUMAN Keratin, type I cytoskeletal 14 OS=Homo sapiens OX=9606 GN=KRT14 PE=1 SV=4	2.43	2.43	426.40	707387.8	1	1	0	1.91	472	204009
7	>sp|Q61414|K1C15_MOUSE Keratin, type I cytoskeletal 15 OS=Mus musculus OX=10090 GN=Krt15 PE=1 SV=2										
7	>sp|Q6IFV3|K1C15_RAT Keratin, type I cytoskeletal 15 OS=Rattus norvegicus OX=10116 GN=Krt15 PE=1 SV=1										
7	>sp|P08779|K1C16_HUMAN Keratin, type I cytoskeletal 16 OS=Homo sapiens OX=9606 GN=KRT16 PE=1 SV=4										
8	>sp|Q02362|ICP4_GAHVG Major viral transcription factor ICP4 homolog OS=Gallid herpesvirus 2 (strain GA) OX=10388 GN=ICP4 PE=3 SV=1	2.34	2.30	296.70	7735090.7	3	1	0	0.64	1415	190444
8	>sp|Q9DGT6|ICP4_GAHVM Major viral transcription factor ICP4 homolog OS=Gallid herpesvirus 2 (strain Chicken/Md5/ATCC VR-987) OX=10389 GN=MDV084 PE=3 SV=1										
9	>sp|O18740|K1C9_CANLF Keratin, type I cytoskeletal 9 OS=Canis lupus familiaris OX=9615 GN=KRT9 PE=3 SV=1	2.13	2.11	413.60	1921686.7	2	1	0	1.15	786	204084
10	>sp|Q5XQN5|K2C5_BOVIN Keratin, type II cytoskeletal 5 OS=Bos taurus OX=9913 GN=KRT5 PE=3 SV=1	2.09	2.09	443.80	309109.5	1	1	0	1.66	601	204146
10	>sp|Q922U2|K2C5_MOUSE Keratin, type II cytoskeletal 5 OS=Mus musculus OX=10090 GN=Krt5 PE=1 SV=1										
10	>sp|Q6P6Q2|K2C5_RAT Keratin, type II cytoskeletal 5 OS=Rattus norvegicus OX=10116 GN=Krt5 PE=1 SV=1										
11	>sp|Q4FZU2|K2C6A_RAT Keratin, type II cytoskeletal 6A OS=Rattus norvegicus OX=10116 GN=Krt6a PE=1 SV=1	1.84	1.84	344.60	572335.5	1	1	0	1.27	552	204154
12	>sp|P01827|HV2A_RABIT Ig heavy chain V-A2 region BS-1 OS=Oryctolagus cuniculus OX=9986 PE=1 SV=1	1.64	1.64	409.30	474275.7	1	1	0	10.53	114	188482
13	>sp|Q9FIZ7|OPLA_ARATH 5-oxoprolinase OS=Arabidopsis thaliana OX=3702 GN=OXP1 PE=1 SV=1	1.57	1.57	317.70	638817.0	1	1	0	0.63	1266	292577
13	>sp|Q54NW6|OPLA_DICDI 5-oxoprolinase OS=Dictyostelium discoideum OX=44689 GN=oplah PE=3 SV=2										
14	>sp|P98164|LRP2_HUMAN Low-density lipoprotein receptor-related protein 2 OS=Homo sapiens OX=9606 GN=LRP2 PE=1 SV=3	1.42	1.36	300.70	10455448.0	4	1	0	0.17	4655	227706
15	>sp|Q9TLZ2|PSB28_CYACA Photosystem II reaction center Psb28 protein OS=Cyanidium caldarium OX=2771 GN=psb28 PE=3 SV=1	1.13	1.13	298.80	524699.1	1	1	0	7.83	115	326353
16	>sp|B0CR45|NAGS_LACBS Amino-acid acetyltransferase, mitochondrial OS=Laccaria bicolor (strain S238N-H82 / ATCC MYA-4686) OX=486041 GN=ARG2 PE=3 SV=1	1.06	1.06	311.20	589084.9	1	1	0	1.25	559	267566
17	>sp|A4VS60|GLMU_PSEU5 Bifunctional protein GlmU OS=Pseudomonas stutzeri (strain A1501) OX=379731 GN=glmU PE=3 SV=1	1.02	1.02	364.70	636976.2	1	1	0	1.55	452	156004
18	>sp|Q5I147|PTPH1_MDBVW Tyrosine phosphatase-like protein H1 OS=Microplitis demolitor bracovirus (isolate Webb) OX=654919 GN=H1 PE=1 SV=1	0.98	0.96	222.50	1937813.6	2	1	0	3.87	336	332895
19	>sp|A5F465|FADB_VIBC3 Fatty acid oxidation complex subunit alpha OS=Vibrio cholerae serotype O1 (strain ATCC 39541 / Classical Ogawa 395 / O395) OX=345073 GN=fadB	0.93	0.91	240.30	1494179.3	2	1	0	1.11	723	130520
19	>sp|Q9KNI1|FADB_VIBCH Fatty acid oxidation complex subunit alpha OS=Vibrio cholerae serotype O1 (strain ATCC 39315 / El Tor Inaba N16961) OX=243277 GN=fadB PE=3 S										
19	>sp|C3LSI3|FADB_VIBCM Fatty acid oxidation complex subunit alpha OS=Vibrio cholerae serotype O1 (strain M66-2) OX=579112 GN=fadB PE=3 SV=1										
19	>sp|Q8DDK6|FADB_VIBVU Fatty acid oxidation complex subunit alpha OS=Vibrio vulnificus (strain CMCP6) OX=216895 GN=fadB PE=3 SV=1										
19	>sp|Q7MQH9|FADB_VIBVY Fatty acid oxidation complex subunit alpha OS=Vibrio vulnificus (strain YJ016) OX=196600 GN=fadB PE=3 SV=1										

(B)

>sp|P08572|CO4A2_HUMAN Collagen alpha-2(IV) chain OS=Homo sapiens OX=9606 GN=COL4A2 PE=1 SV=4

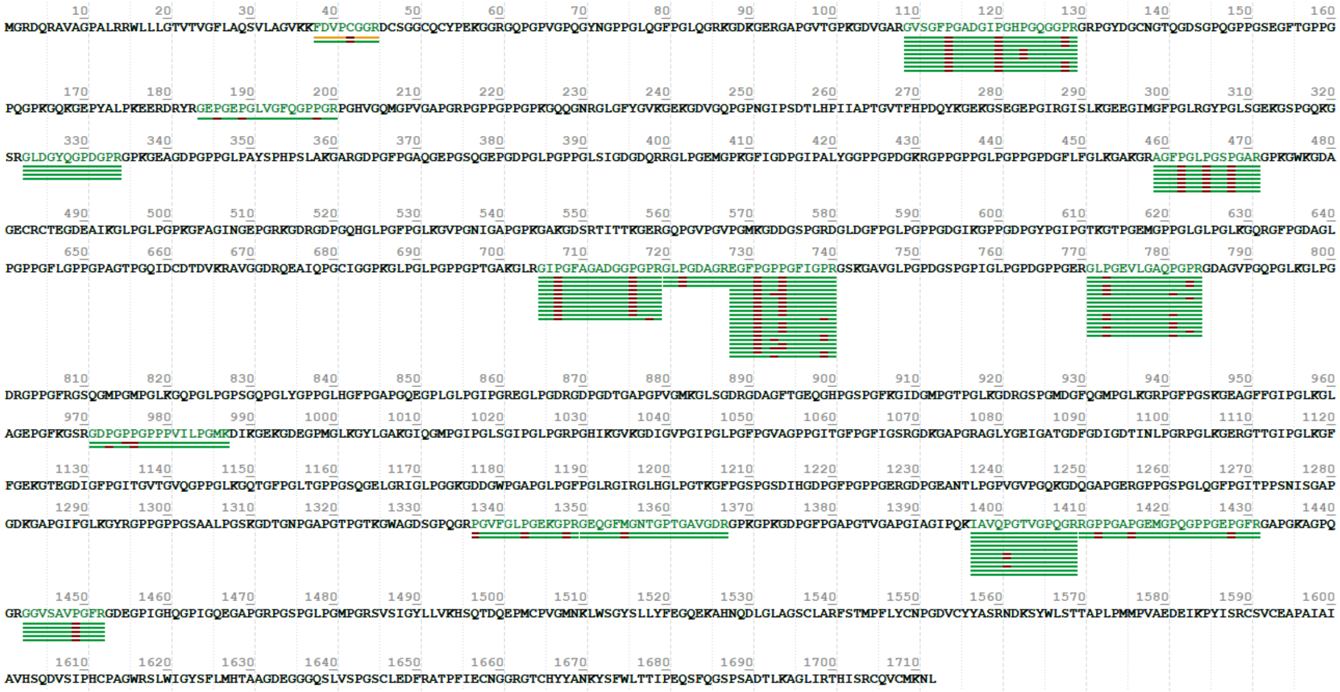

Supplementary Fig. 3

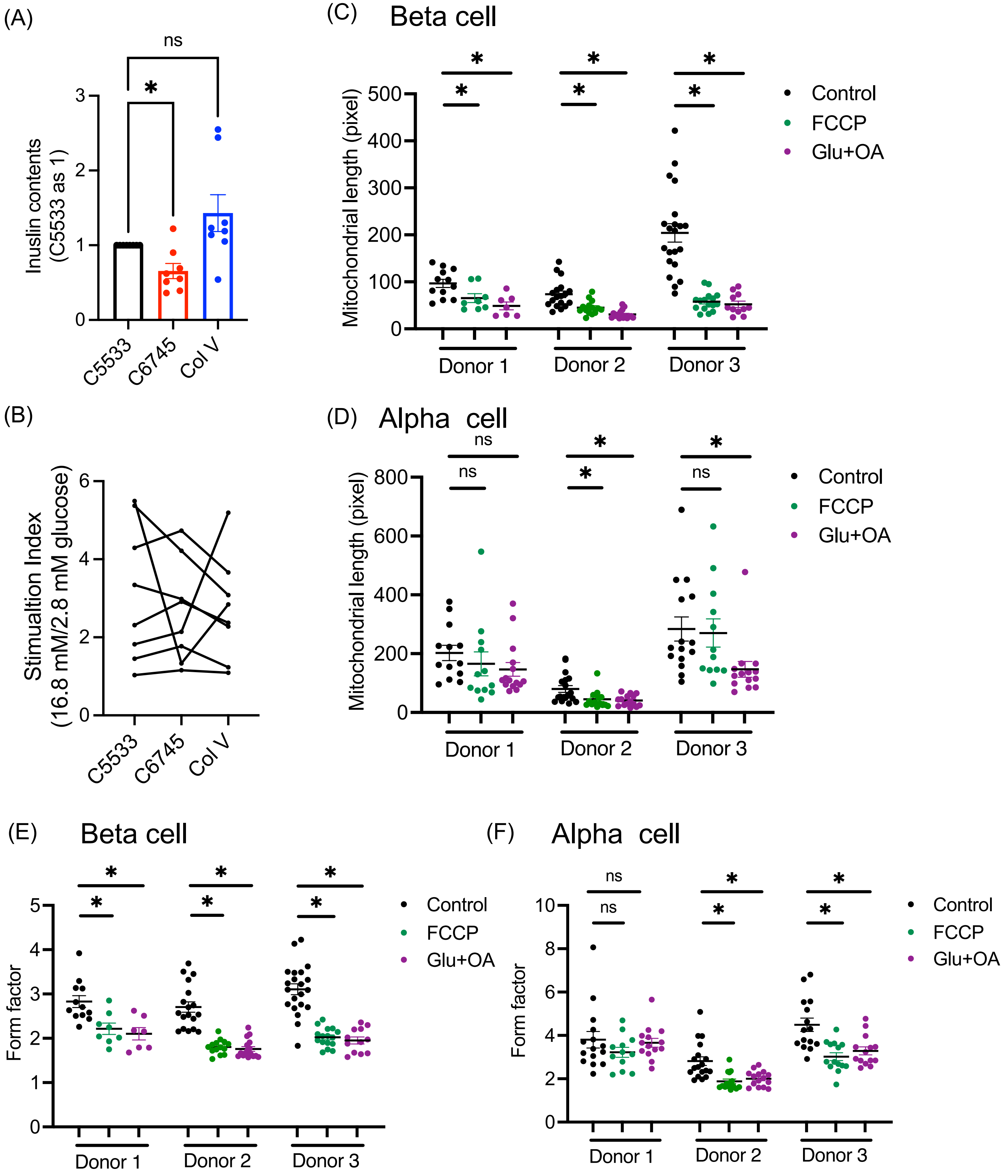

Supplement: Supplementary file 1 [file Presentation_1.pdf]
